# Cloning and expression study of a high-affinity nitrate transporter gene from *Zea mays L*

**DOI:** 10.1080/15592324.2022.2163342

**Published:** 2023-01-16

**Authors:** Guoliang Li, Xu Chang, Yi Dong, Mingquan Wang, Jianfei Yang, Guanghui Hu, Jin Shumei

**Affiliations:** a Heilongjiang Academy of Agricultural Sciences, Harbin, Heilongjiang, China; b Key Laboratory of Saline-Alkali Vegetation Ecology Restoration, Ministry of Education, College of Life Sciences, Northeast Forestry University, Harbin, Heilongjiang, China; c Aulin College, Northeast Forestry University, Harbin, Heilongjiang, China

**Keywords:** *Zea mays*, High-affinity nitrate transporter gene, salt shock

## Abstract

A nitrate transporter gene, named *B46NRT2.1*, from salt-tolerant *Zea mays* L. B46 has been cloned. B46NRT2.1 contained the same domain belonging to the major facilitator superfamily (PLN00028). The results of the phylogenetic tree indicated that B46NRT2.1 exhibits sequence similarity and the closest relationship with those known nitrate transporters of the NRT2 family. Through RT-qPCR, we found that the expression of *B46NRT2.1* mainly happens in the root and leaf. Moreover, the treatment with NaCl, Na_2_CO_3_, and NaHCO_3_ could significantly increase the expression of *B46NRT2.1*. B46NRT2.1 was located in the plasma membrane. Through the study of yeast and plant salt response brought by *B46NRT2.1* overexpression, we have preliminary knowledge that the expression of *B46NRT2.1* makes yeast and plants respond to salt shock. There are 10 different kinds of cis-acting regulatory elements (CRES) in the promotor sequences of *B46NRT2.1* gene using the PlantCARE web server to analyze. It mainly includes hormone response, abscisic acid, salicylic acid, gibberellin, methyl jasmonate, and auxin. The *B46NRT2.1* gene’s co-expression network showed that it was co-expressed with a number of other genes in several biological pathways, including regulation of NO_3_ long-distance transit, modulation of nitrate sensing and metabolism, nitrate assimilation, and transduction of Jasmonic acid-independent wound signal. The results of this work should serve as a good scientific foundation for further research on the functions of the *NRT2* gene family in plants (inbred line B46), and this research adds to our understanding of the molecular mechanisms under salt tolerance.

## Introduction

As the largest crop in China, in the past 20 years, the planting area and yield of maize in China have increased by 170% and 228%, respectively. The growth and productivity of maize (*Zea mays L*.) are impacted by salinity. Numerous salt-related genes in maize have been investigated in order to better understand the molecular processes of resistance to salinity treatment in maize. Through a molecular mechanism linked to hormone signaling, ROS scavenging, and root hair flexibility, *ZmEREB20* (Ethylene Responsive Element Binding 20) improved salt tolerance.^1^ Through an ABA-dependent mechanism, *ZmMYB3R* (*Zea mays L*. v-myb avian myeloblastosis viral oncogene homolog 3 R) enhances tolerance to salt treatment.^2^ Overexpression of *ZmBSK1* (*Zea mays L*. brassinosteroid-signaling kinase 1) in maize increased the salt tolerance.^3^ Overexpression of *ZmCIPK21* (*Zea mays L*. calcineurin B-like protein-interacting protein kinase 21) in *Arabidopsis* improved salt tolerance.^4^ Under salt conditions, *Arabidopsis* overexpressing *ZmbHLH55* (*Zea mays L*. basic helix-loop-helix 55) showed higher AsA (Ascorbic acid) levels, quicker germination, and enhanced antioxidant enzyme activity.^5^ *ZmGPDH1* (*Zea mays L*. glycerol-3-phosphate dehydrogenase) modulates glycerol production, stomatal closure, cellular redox, and ROS homeostasis in *Arabidopsis* to enable it to tolerate salt.^6^ By modulating sodium and potassium homeostasis and maintaining photosystem II, *ZmCPK11* (*Zea mays L*. calcium-dependent protein kinase) increases salt tolerance in transgenic *Arabidopsis* plants.^7^ A total of 296 genes were precisely comparative and transcriptionally regulated from the roots and leaves of three-leaf stage seedlings of the maize inbred line YQ7-96 under circumstances of 100 mM NaCl and salt removal.^8^ Microarray-based studies of maize gene expression under salt shock were carried out in maize roots.^9^

For a plant to grow and develop, nitrogen must be transported via inorganic nitrate. Nitrate transporters are essential for transporting nitrate. In plants, NO_3_ is frequently transferred by high-affinity transport systems (HATS) and low-affinity transport systems (LATS). The NRT1 and NRT2 gene families have been found to encode nitrate transporters.^10^ NRT1 and the NRT2 family are categorized as LATS and HATS, respectively, since higher plants’ high-affinity NO_3_-transporters are divided into two groups: iHAT (inducible high-affinity NO_3_-transporter) and cHAT (constitutive high-affinity NO_3_-transporter).^11^ Four homologs of AtNPF6.3 (iHAT) have been identified in maize. NO_3_ transport activity was demonstrated by ZmNRT6.4.^9^ Substantial spatiotemporal expression analysis of *NRT2.1* has previously shown the molecular processes behind this high-affinity NO_3_^−^ transport pathway in Arabidopsis.^12^ Gene encoding NRT2.1 proteins have been found and functionally defined in maize. Four *NRT2* transcripts were previously identified in maize genome.^13^ *ZmNRT2.1* (NCBI GenBank Accession number AY129953), which is the first nitrate transporter found in maize, the effect of nitrate supply on the root of etiolated maize seedlings grown in hydroponics.^14^ The treatment induced higher uptake rates of the anion and the expression of *ZmNRT2*.1.^15^ The expression of *ZmNRT1.1, ZmNRT2.1, ZmNRT2.2*, and *ZmNAR2.1* was stronger and lasted for a longer time after NO_3_ induction.^16^ *ZmNRT2.1* exhibit altered responses of growth and gene expression to nitrate and calcium.^17^ An oligomer composed by two *ZmNRT2.1* and two *ZmNRT3.1A* might be involved in the NO_3_^−^ uptake in maize roots upon induction.^18^ In addition, transcriptional studies of maize treated with saline-alkali treatment revealed that *NRT2.1* was significantly up-regulated.^19^ To study the *NRT2.1* function related to salt, it is important to clone this gene and invest the gene expression patterns in response to salt. Moreover, observing the different physiological indicators between the wildtype and overexpression lines is also necessary.

## Materials and methods

### Material of plants and growth conditions

In this study, *Zea mays* L. B46 (salt-tolerant inbred line) and *Arabidopsis thaliana* (Col-0) were employed. They were grown in an artificial climate incubator with a relative humidity of 70%, dark for 12 hours at 18°C and light for 12 hours at 25°C.

### *The cloning of gene* NRT2.1 *from* Zea mays *L*

Total RNA was extracted from *Zea mays* L. B46 using the RNeasy Plant MiniKit (Qiagen, Hilden, Germany), and the cDNA was generated using the reverse transcription PCR kit (Takara, Japan’s Tokyo) from the total RNA. The specific primers (*B46NRT2.1 F*: 5’-ATGGCGGCCGTCGGCGCTC-3’ and *B46NRT2.1 R*: 5’-TTAGACATGCTCCGGCGT-3’) were designed by analyzing the transcriptome sequence of B46 lines, the amplified PCR product was sequenced.

### The analysis of conserved domain of B46NRT1.1

NCBI (National Center for Biotechnology Information) examined the conserved domain sequence of B46NRT1.1 and the amino acid sequence was highly similar to other species. DNAMAN software was used to compare the protein’s homologous amino acid sequence, and MEGA7 was used to construct the phylogenetic tree.

### *RT-qPCR analysis of* B46NRT2.1 *expression in* Zea mays

Total RNA was extracted from *Zea mays L*. of different organs, RT-qPCR was carried out in IQ5 real-time PCR equipment (Bio-Rad, Hercules, CA, USA) with SYBR green (Takara, Tokyo, Japan). The primers were designed by SNAPGENE (*B46NRT2.1qRTF*: ATCATCCGCGACAACCTCAA and *B46NRT2.1qRTR*: AACCTGACGGTGATGTAGCC). As an internal reference, the expression of the *Maize18S* gene was examined. The primer sequence were *Maize18SF*:5’-CAACCATAAACGATGCCGA-3’ and *Maize18SR*: 5’-AGCCTTGCGACCATACTCC-3’.

*Zea mays* L. B46 was tested for *B46NRT2.1* expression patterns under salt shock. The seeds was sowed onto the 1/2 MS medium. The 14-day seedlings were given treatments (300 mM NaCl, 100 mM Na_2_CO_3_, or 150 mM NaHCO_3_) for 0, 6, 12, 2, 4, or 36 hours. The expression of *B46NRT2.1* in the seedlings under different treatments were detected by RT-qPCR.

### Construction of expression vectors and transformation

The open reading frame (ORF) of the *B46NRT2.1* was ligated to the pBI121-35SGFP, pYES2, pGEX-6p-3 and pBI121 vector using infusion cloning, respectively. It was finally verified by sequenced.

### *Subcellular localization of* B46NRT2.1 *in transgenic* Nicotiana benthamiana

For the transient expression in *N. benthamiana, Agrobacterium* strains GV3101 carrying pBI121-*35SGFP-B46NRT2.1*, pBI121-*35SGFP* infiltrated the abaxial side of the leaves of *N. benthamiana* seedlings that were four weeks old. All plants were kept in the culture chamber for two days after infiltration. The lower epidermis of the leaf was photographed under a 488 nm GFP argon laser using confocal two-photon laser scanning mode (TCS SP8 MP, Leica, Germany).^20^

### Salt shock response experiments

The *E. Coli* cells BL21 with pGEX-6p-3-*B46NRT2.1* were used for the expression of the B46NRT2.1 fusion protein, and the *E. Coli* cells with pGEX-6p-3 were used as the control. In LB liquid medium at 37°C, the bacteria solution *pGEX-6p-3* and *pGEX-6p-3-B46NRT2.1* were cultured until the OD_600_ = 0.5. After being exposed to 0.8 M NaCl, 0.1 M Na_2_CO_3_, or 0.2 M NaHCO_3_ for one hour, the protein was treated with 1 mM IPTG to induce expression. The cultures were cultivated by rotary shaking (160 rpm) at 37°C for 1, 2, 3, 4, and 5 hours. The growth rate of the strains was observed using a spectrometer by measuring changes in absorbance at 600 nm. The data for three duplicate studies are preliminary.

The pYES2-*B46NRT2.1* and pYES2 were transformed into the yeast (*Saccharomyces cerevisiae*) strain *INVSC1* (Takara, Tokyo, Japan) following the manufacturer’s instructions (Invitrogen). The strain *INVSC1* with the pYES2-*B46NRT2.1* and pYES2 were cultivated in YPD medium (Yeast extract peptone dextrose) overnight at 30°C. After the culture solutions concentration reached OD_600_ = 0.6, the solutions with repeated dilutions (10^−1^, 10^−2^, 10^−3^ and 10^−4^) were dripped onto YPD agar plates with no treatment (CK), 30 mM NaHCO_3_, 1 M NaCl, or 10 mM Na_2_CO_3_.

*Arabidopsis Thaliana* (*A. Thaliana*) was infected using *Agrobacterium tumefaciens* strain *EHA105* (Takara, Tokyo, Japan) carrying plasmid pBI121-*B46NRT2.1* by floral dip method.^20^ *A. thaliana* lines seeds of the T3 generation homozygous overexpression and wild types were raised on 1/2 MS medium with no treatment (CK), 100 mM NaCl, 125 mM NaCl, 150 mM NaCl, 3 mM Na_2_CO_3_, 5 mM Na_2_CO_3_, 7 mM Na_2_CO_3_, 3 mM NaHCO_3_, 5 mM NaHCO_3_ or 7 mM NaHCO_3_. After 14 days, the experiment was repeated in triplicate and separately photographed.

Two-week-old seedlings of similar size were placed on 1/2 MS medium supplemented with no (CK) and a variety of treatment (200 mM NaCl; 20 mM Na_2_CO_3_ or 20 mM NaHCO_3_), respectively. Each group had the previously indicated three replications. The Petri plates were positioned vertically to view the root development. After receiving a 7-day treatment, plants were photographed.

### *Analysis of* B46NRT2.1 *promoter sequence*

The *B46NRT2.1* gene was located in chromosome 4 (B73 RefGen_v3: Chr4:240778577.240782331) of *Zea may* analyzing by the maize database (https://maizegdb.org/). The 2000 bp region upstream of the initiation codon (ATG) of the *NRT2.1* gene was used to examine the promoter sequences of the *B46NRT2.1* gene. They were obtained from the database (https://maizegdb.org/) and submitted to the plantcare server (http://bioinformatics.psb.ugent.be/webtoo ls/plantcare/html/).

### B46NRT2.1 interacting protein analysis

Through the maize genome website (https://maizegdb.org/), NRT2.1 protein co-expression network was analyzed using the TomExpress platforms.

### Statistical analysis

All data are presented as mean ± standard Error (SE), n = 3. Significant differences were analyzed by T test by GraphPad Prism 8 software.

## Results

### *Cloning and sequence analysis of* B46NRT2.1

**Figure 1.**
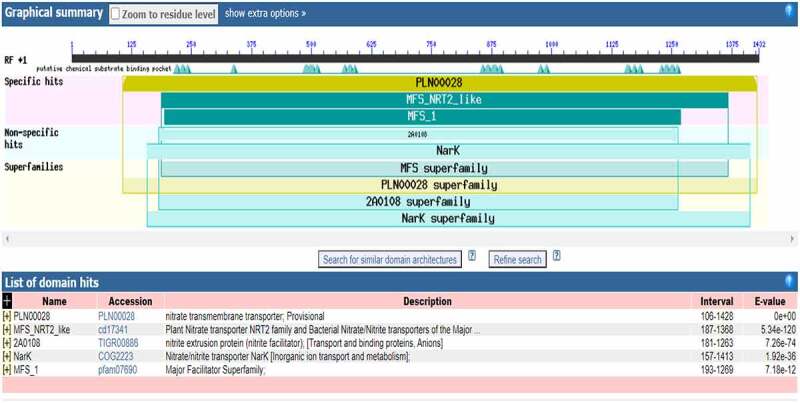
The analysis of conserved domain of *B46NRT2.1*. The gene *B46NRT2.1* mainly contains specific hits (PLN00028, MFS_NRT2_like, MFS_2), nonspecific hits (2A0108, NarK), and superfamilies (MFS superfamily, PLN00028, 2A0108 superfamily, NarK superfamilies).

**Figure 2.**
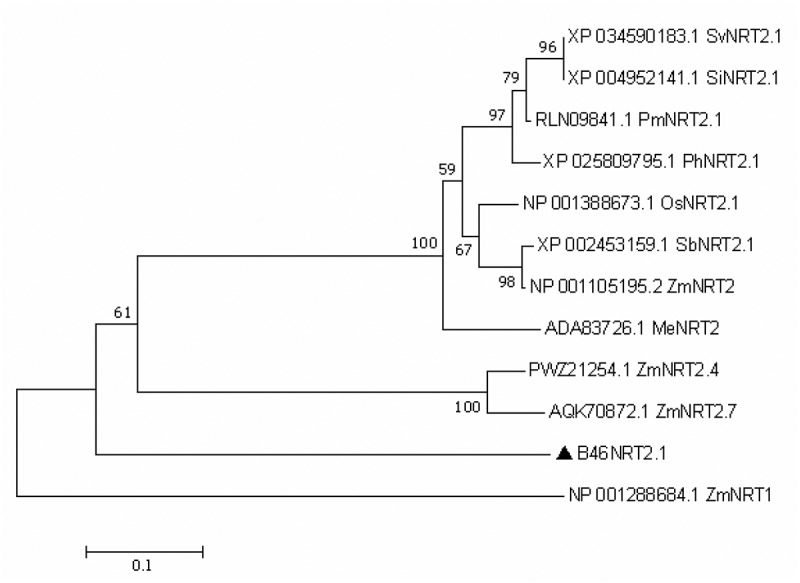
Phylogenetic tree of the B46NRT2.1 using MEGA7 software. The bar represents 0.1 aa substitution for pre site. (Sv: *Setaria viridis*; Si: *Setaria italica*; Pm: *Panicum miliaceum*; Ph: *Panicum hallii*; Os: *Oryza sativa*; Sb: *Sorghum bicolor* Me: *Manihot esculenta*).

**Figure 3.**
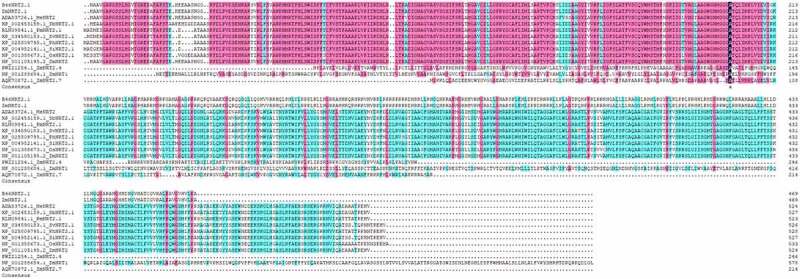
The alignment of the B46NRT2.1 amino acid sequence with NRT2.1 proteins from other species. (Sv: *Setaria viridis*; Si: Setaria italica; Pm: *Panicum miliaceum*; Ph: *Panicum hallii*; Os: *Oryza sativa*; Sb: *Sorghum bicolor* Me: *Manihot esculenta*;)

A DNA fragment (supplemental file) with 1410 bp was obtained. The ORF sequence of *B46NRT2.1* was encoded 469 amino acids that included the NRT2.1 family’s conserved domains. It presented a high proportion of identification with the PLN00028 (transporters) belonging to NRT2 family (Figure 1). The result of phylogenetic tree analysis was revealed in Figure 2. The alignment of the B46NRT2.1 amino acid sequence was the same with the sequence of ZmNRT2.1 in NCBI. Due to its similarity to previously known sequences, this sequence was named B46NRT2.1. (Figure 3).

### *RT-qPCR analysis for* B46NRT1.1 *expression in* zea mays

**Figure 4.**
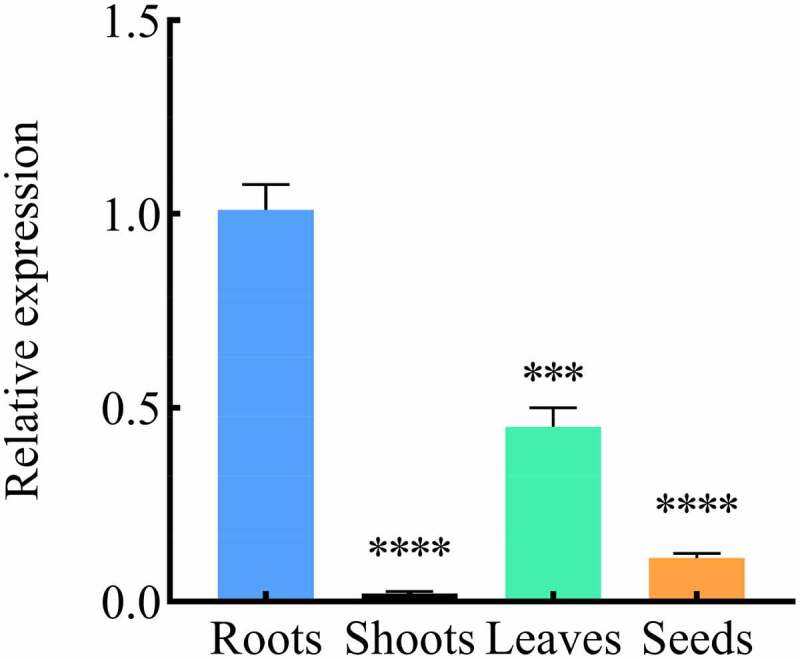
The expression level of the *B46NRT2.1* in different organs were examined using RT-qPCR. cDNA was obtained from different organs (Roots, Shoots, Leaves, Seeds) of *Zea mays* L.B46 lines, and the transcript abundances was detected by RT-qPCR to investigate *B46NRT2.1* expression in the different organs of *Zea mays* L.B46 lines. ***Significance P < .001, ****Significance P < .0001.

The expression of *B46NRT2.1* may affect the salt response of *Zea mays* L.B46. The expression level of *B46NRT2.1* in various organs of *Zea mays* L. was the highest in the roots, following by leaves, seeds, and shoots (Figure 4).

**Figure 5.**
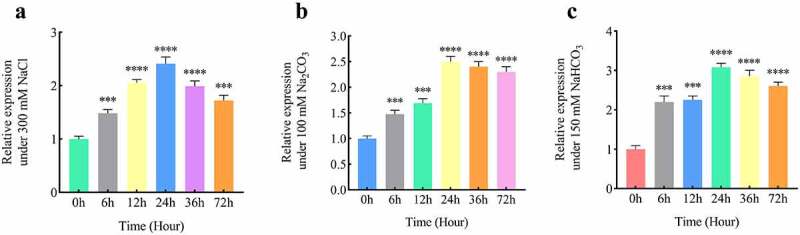
RT-qPCR analysis of *B46NRT1.1* expression level of *Zea mays* L. under different salt shock. B46 lines were treated with 300 mM NaCl (a), 100 mM Na_2_CO_3_ (b), or 150 mM NaHCO_3_ (c) for 0 h, 6h, 12 h, 24 h, 36 h, and 72 h respectively, then cDNA was obtained from *Zea mays* L. with different salt treatment, and the transcript abundances was detected by RT-qPCR to investigate the impact of salinity on *B46NRT2.1* expression.

The expression of *B46NRT2*.1 increased gradually and peaked at 24 hours under conditions of 300 mM NaCl, 100 mM Na_2_CO_3_ or 150 mM NaHCO_3_, which are nearly 2.3 times higher than untreated under 300 mM NaCl (Figure 5 (a)), almost 2.7 times higher than untreated under 100 mM Na_2_CO_3_ (Figure 5(b)), over 3.1 times higher than untreated under 150 mM NaHCO_3_ (Figure 5(c)).

### Subcellular localization of B46NRT2.1

The pBI121-*GFP-B46NRT2.1* and the control pBI121-*GFP* were transiently transformed into leaf cells of *N. benthamiana* via agroinfiltration to ascertain the subcellular location of B46NRT2.1. The pBI121-GFP protein was discovered in the whole cell (Figure 5(a)). The pBI121-GFP-B46NRT2.1 protein was discovered in the plasma membrane (Figure 5(b)).

### The expression analysis of B46NRT2.1 in E. coli under salt shock

**Figure 6.**
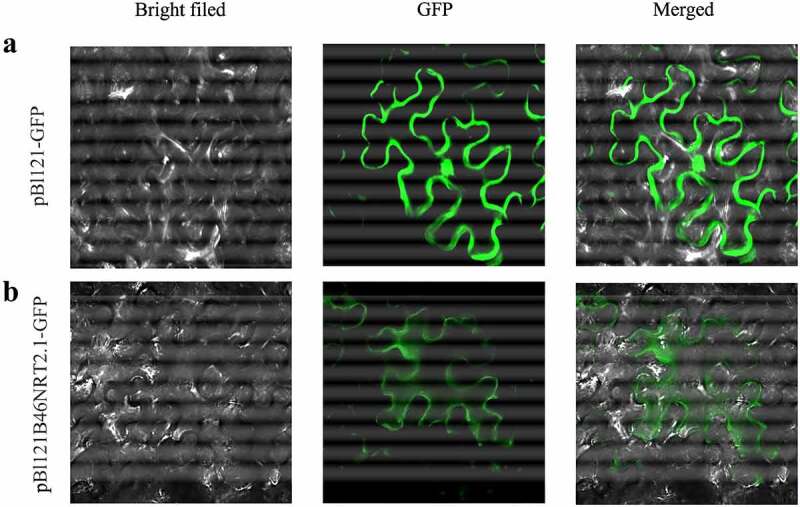
Subcellular localization of the B46NRT2.1. Imaging was captured respectively visualized under bright channel (Bright), green fluorescent protein channel (GFP), and merged GFP and bright light channel (GFP + Bright) in *N. benthamiana* cells. A. Expression of the GFP protein in *N. benthamiana*. B. Expression of the B46NRT2.1-GFP fusion protein in *N. benthamiana.*

The growth of *E. coli* with the pGEX vector or pGEX-*B46NRT2.1* were compared in order to examine the salt response of *B46NRT2.1* expression in *E. coli*. There is no difference between *E. coli* with pGEX vector and *E. coli* expressing pGEX-*B46NRT2.1* in growth with no salt treatment (Figure 6(a)). The control strain’s OD_600_ values dropped to 0.57 after being exposed to 0.8 M NaCl, however the transgenic strain’s values remained unchanged after 1 h of incubation. After 5 hours of culture, the transgenic strain’s and the control strain’s OD_600_ values were recorded as 1.38 and 1.62, respectively (Figure 6(b)). The OD_600_ values of the control and transgenic strains were 0.29 and 0.48 after 1 h of treatment with 0.1 M Na_2_CO_3_. The OD_600_ values were 0.69 and 1.45, respectively, after 5 hours (Figure 6(c)). Control and transgenic strains were cultivated with 0.2 M NaHCO_3_ treatment, and after 1 h, their OD_600_ values were 0.41 and 0.65, respectively. After five hours, these values were 0.98 and 1.67, respectively. (Figure 6(d)). Under the saline shock, the *E. coli*. cell containing pGEX-*B46NRT2.1* had a better growth rate compared with the *E. coli*. cell containing pGEX.

### B46NRT2.1 *transgenic yeast response to salinity*

**Figure 7.**
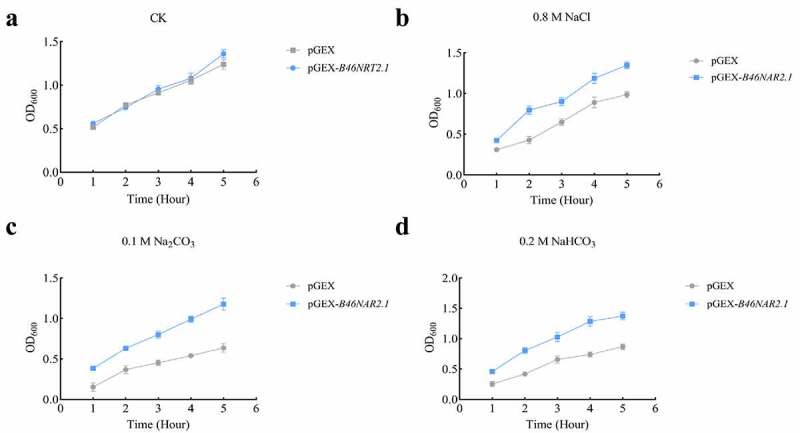
The growth rate of *E. coli* cells with the expression of *B46NRT2.1* or not under different salt treatment. The protein expression strain BL21 was transformed by PGEX vector or pGEX-*B46NRT2.1* plasmid. These *E. coli* cells were induced by 1 mM IPTG for 1,2,3,4,5 h under different salt treatment, and then the growth rate of them were measured using spectrometer (OD_600_). A. No salt treatment (CK). B. 0.8 M NaCl treatment. C. 0.1 M Na_2_CO_3_ treatment. D. 0.2 M NaHCO_3_ treatment.

*B46NRT2.1* expression in the yeast was investigated. The growth of the yeast cell with pYES2-*B46NRT2.1* or pYES2 were compared under different salt treatments (Figure 7). The yeasts carrying the pYES2 or pYES2-*B46NRT2.1* plasmid grew similarly (upper and lower lines, respectively) under no salt treatment. The yeasts carrying pYES2-*B46NRT2.1* developed considerably faster than that of control under 30 mM NaHCO_3_, 1 M NaCl, and 20 mM Na_2_CO_3_ treatments. The result indicated that *B46NRT2.1* expression had improved yeast growth than control under saline shock.

## *Identification of* B46NRT2.1 *transgenic* A. thaliana

**Figure 8.**
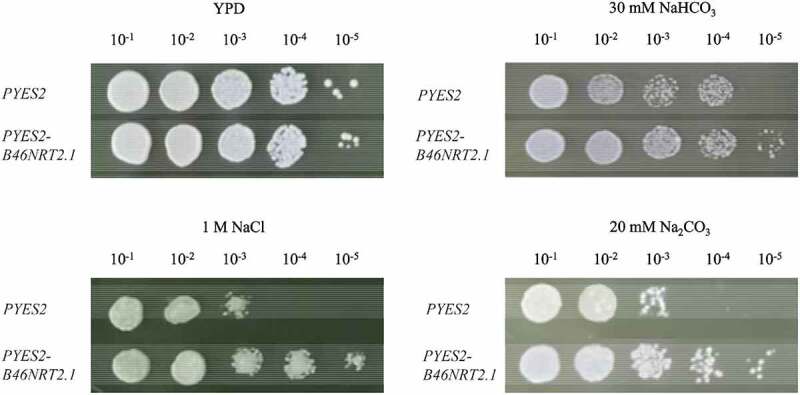
The growth of yeast cells carrying the pYES2 or pYES2-*B46NRT2.1* under salt shock. Ten-fold dilutions of yeast cells containing pYES2 (upper line) and pYES2-*B46NRT2.1* (lower line) were spotted onto solid YPG media supplemented with the 30 mM NaHCO_3_, 1 M NaCl, and 20 mM Na_2_CO_3_ treatment. No treatment was used as a control with YPD media.

RT-qPCR was used to identify the expression of *B46NRT2.1* in overexpression *A. thaliana*. (Figure 8). Six *B46NRT2.1* overexpression *A. thaliana* lines were chosen at random, and all of them had higher levels of *B46NRT2.1* expression than the wild type. *B46NRT2.1* expression was 22, 28, 21, 20, 24, and 14 times greater in the overexpression lines (OTL1-OTL6) than in wild-type plants, respectively.

### The analysis of *B46NRT2.1* overexpression *A. thaliana* under salt shock

**Figure 9.**
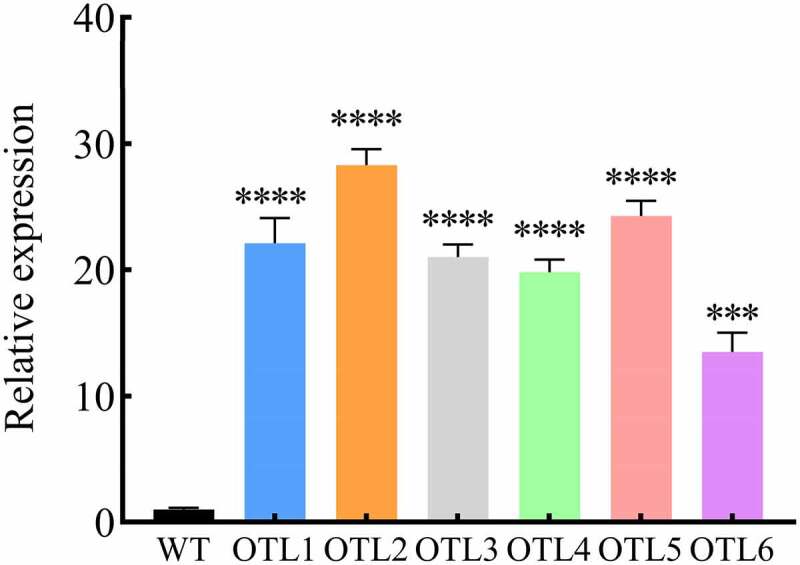
Identification of *B46NRT2.1* overexpression *A. thaliana* lines. The expression contrasting of *B46NRT2.1* in the wild-type and overexpression lines were measured using RT-qPCR. WT: Wide type. OTL1-6: *B46NRT2.1* transgenic *A. thaliana* lines.

When overexpression plant seeds are directly sown on 1/2 MS, there is no difference between them and wild-type plants (CK). Overexpression plant seeds had larger leaves than wild-type plants when treated with 100 mM NaCl, 3 mM NaHCO_3_, or 5 mM NaHCO_3_, although germination rate was equivalent. On the medium containing 125 mM NaCl, 150 mM NaCl, 3 mM Na_2_CO_3_, 5 mM Na_2_CO_3_, 7 mM Na_2_CO_3_ or 7 mM NaHCO_3_, overexpression plant seeds germinated 1–2 days sooner than wild-type seeds. Moreover, the seedlings of wild-type *A*. th*aliana* were light-colored, and leaves were severely curled than that of overexpression lines (Figure 9). The overexpression *A. thaliana* lines’ seeds all grew into fully grown plants, all of which were green. Although both wild and overexpression plants responded to salt shock, the overexpression plants suffered less damage than the wild-type plants.

Additionally, *B46NRT2.1* overexpression line and wild type were grown at the seedling stage on 1/2 MS medium without treatment (CK) or treatment for two weeks (Figure 9). Under typical growing conditions, neither the *B46NRT2.1* overexpression lines nor the wild-type seedlings displayed any obvious morphological or developmental flaws. In the wide-type, additional NaCl treatment caused the leaf edges to brown and the color to darken. When wild-type seedlings were cultivated on 1/2 MS media with Na_2_CO_3_, their cotyledons were smaller than those of *B46NRT2.1* overexpression line and the majority of wild-type leaves became white (Figure 9). The result indicated that *B46NRT2.1* overexpression plants had less effect than wildtype to salt shock.

**Figure 10.**
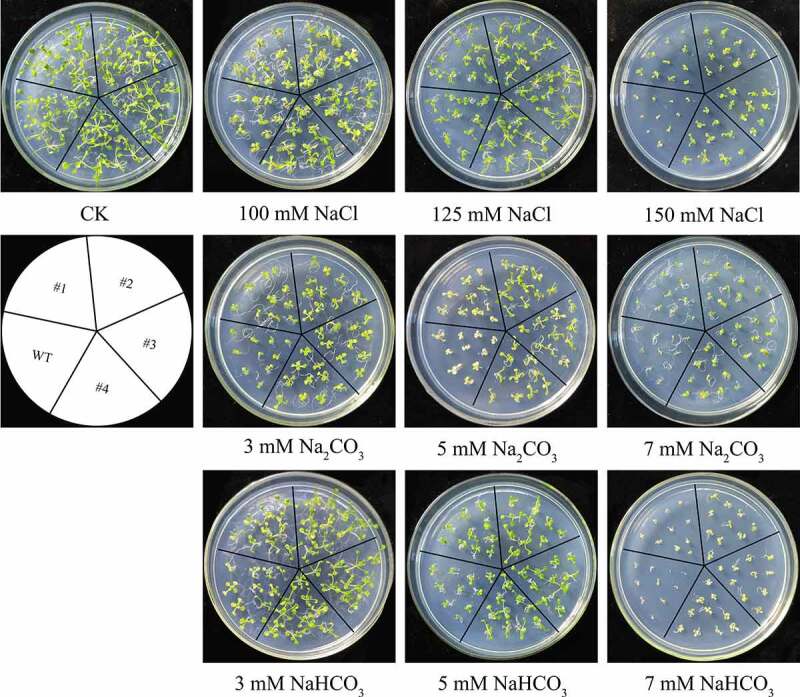
Seed germination in *A. thaliana* wild-type and *B46NRT2.1* overexpression plants under different stresses. Seed of T3 overexpression *A. thaliana* and wild-type were grown on 1/2 MS medium supplemented with 0 mM (CK); 100 mM NaCl, 125 mM NaCl, 150 mM NaCl; 3 mM Na_2_CO_3_, 5 mM Na_2_CO_3_, 7 mM Na_2_CO_3_; 3 mM NaHCO_3_, 5 mM NaHCO_3_, or 7 mM NaHCO_3_, respectively. WT: Wild-type *A. thaliana*. #1, #2, #3 and #4: *B46NRT2.1* overexpression *A. thaliana* lines.

There is no difference of seeding length between wild type and overexpression plant with no treatment (CK). Salinity showed a negative influence on the leaf of plant. However, compared with WT, the leaves are less wilting in overexpression lines (Figure 10).

### *The cis-acting elements analysis of* B46NRT2.1 *gene*

**Figure 11.**
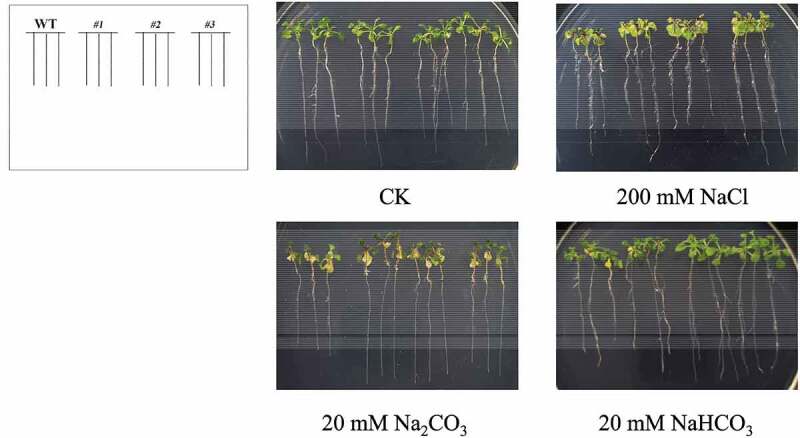
The phenotype of the WT and overexpression *Arabidopsis* lines seedlings under different stresses. The sterilized *Arabidopsis* seedlings were evenly plated on 1/2 MS with 200 mM NaCl, 20 mM Na_2_CO_3_, 20 mM NaHCO_3,_ respectively. No treatment (CK) was used as a control. WT: Wild-type *A. thaliana*. #1, #2 and #3: *B46NRT2.1* overexpression lines.

The promotor sequences of *B46NRT*2.1 gene (supplemental file) were analyzed using the PlantCARE web server to get CRES of the *B46NRT2.1* gene. (Figure 11). There are 10 different types of CRES, and these controlling factors primarily consist of auxin, gibberellin, methyl jasmonate, abscisic acid, salicylic acid, and hormone response.

**Figure 12.**
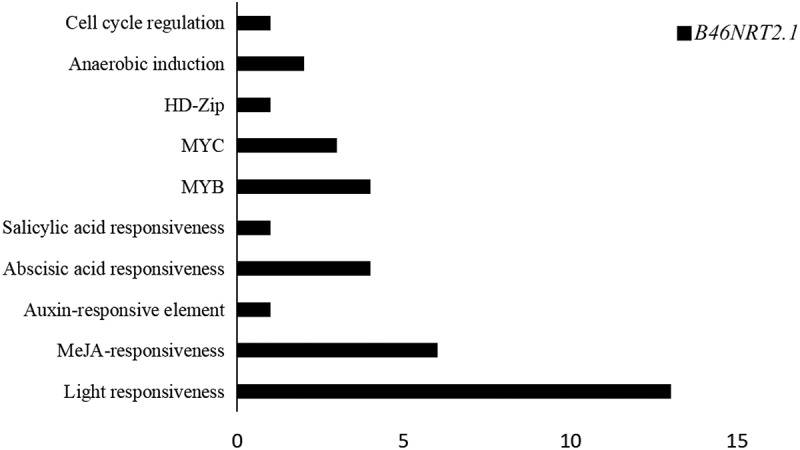
Predicted cis elements in the promoter of the *B46NRT2.1* gene. The 2000 bp region upstream of the initiation codon (ATG) of the *NRT2.1* gene was analyzed to examine the promoter sequences by the PlantCARE online server.

Co-expression network study for B46NRT2.1 protein was carried out using the TomExpress platforms (Figure 12). The results indicated there were 10 proteins had correlation with B46NRT2.1, which has identified and annotated (NRT1.8, NRT1.5, WR3, NLP7, NIA1, NIR1, AMT2, NRT1.1, NIA2, AMT1:1). These proteins including regulation of NO_3_ long-distance transit, modulation of nitrate sensing and metabolism, nitrate assimilation, wrinkled leaf and transduction of Jasmonic acid-independent wound signal.

## Discussion

Alkalinized soils are found all throughout the world, more than 70% of the land in northeast China is alkaline.^21^ The alkali treatment is likely to inhibit NO_3_^−^ uptake and assimilation in plants.^22^ Enhanced N absorption and assimilation boosted plant tolerance to alkali treatment.^23^Salt shock is an extreme form of salt stress, where plants are exposed suddenly to a high level of salinity.^24^ In this study, we chose salt shock to know whether the expression of *NRT2.1* gene is related to salt or not. In addition, further experiments that are gradually exposed to the increasing salt stress will be done to simulate the real situation of agriculture in the future.^25^

Nitrate transporters promote the absorption and transportation of nitrate from the root to other organs. As a high affinity nitrate transporter, the expression of *NRT2.1* is up regulated by nitrate.^26^ Whether *NRT2.1* involvement in salinity conditions was investigated in this work.

The *NRT2.1* gene was cloned from *Zea mays L*. inbred line B46. *B46NRT2.1* possesses all the standard characteristics of the HATS-type NRT2.^10^ The evolutionary relationship between NRT in different plants found that *B46NRT2.1* had the highest identity with the *NRT2.1* of other plants.

*B46NRT2.1* was predominantly expressed in the roots, the root system of plants plays an important role in salt response.^27^ When examining crop salt response, root hair is an important factor to take into account since it benefits plants by increasing the surface area that can absorb water and nutrients.^28^ *B46NRT2.1* also expressed in the leaves and shoots which are consistent with *NRT2.1* expression in other plants. *ZmNRT2.1* not only respond for absorption in root but also nitrate assimilation.^29^ Eight different barley organs were found to have low levels of *HvNRT2* gene expression.^30^ The root, flower, leaf, and pericarp tissues of rapeseed were found to have higher expression levels of *BnNRT2* genes.^31^ *PtNRT2* were shown to be more expressed in poplar leaves, wood, and root tissues.^32^

**Figure 13.**
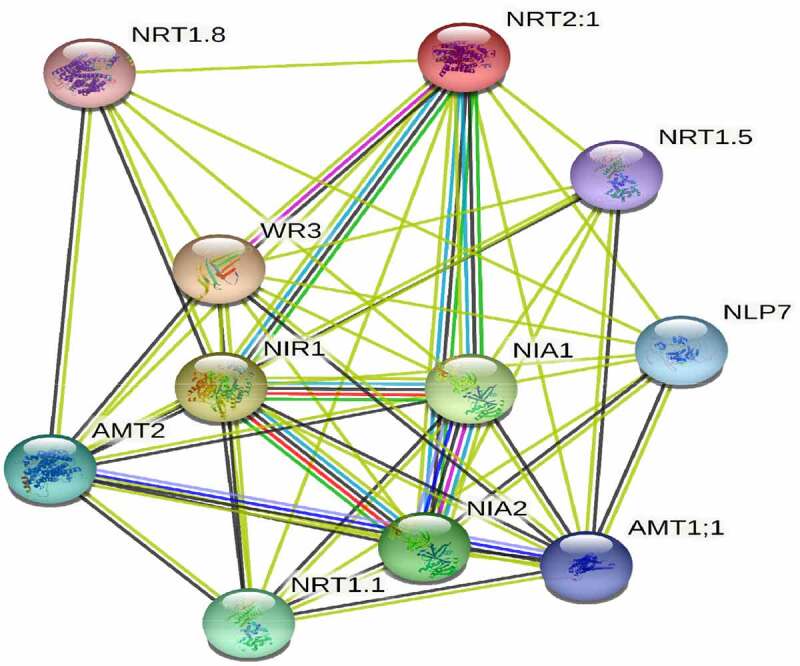
Co-expression network analyses of B46NRT2.1. Co-expression network analyses of B46NRT2.1 using the TomExpress platforms.

The *B46NRT2.1* expression was increased under 300 mM NaCl, 100 mM Na_2_CO_3_ or 150 mM NaHCO_3_ at 24 or 48 h in inbred line B46, respectively (Figure 13). When abele (*Populus tremula alba*) is treated with 75 mM NaCl for one week, the expression of NRT2.1 is enhanced; however, the promotion is abolished after two weeks.^33^ Under salinity treatment, the expression of *DsNRT2.1* is increased in the halophytic alga *Dunaliella salin*a.^34^

B46NRT2.1ʹs subcellular localization showed that the plasma membrane was where it was expressed. Similar results were also attained in *Cucumis sativus* L. and *Arabidopsi*s.^12,35^

Yeast expressing the *B46NRT2.1* grew better than the control under saline shock, suggesting that *B46NRT2.1* was involved in the salt response in yeast, enabling the transgenic yeast more resistant to salt. Ding et al. reported that *Suaeda physophora*, a euhalophyte, can absorb NO_3_ with high affinities when exposed to NaCl.^36^ In another euhalophyte *Salicornia europaea*, NaCl enhances the rate of NO_3_ absorption.^37^

There is a significant growth difference between the *E. Coli* with B46NRT2.1 and the control group. The *E. Coli* with B46NRT2.1 has an obvious superior growth compared with the control under a series of salt shock treatment. These results indicated that *B46NRT2.1* conferring better growth and salinity resistance to *E. coli* under saline shock.

There are 10 different kinds of CRES in the promoter of *B46NRT2.1*. The abiotic stress responsive elements, development regulatory elements, MYB binding sites, and hormone-related elements are the four different types of CREs found in rapeseed *NRT2* genes.^38^ There is a ABA binding sites within the B46 promoter sequence. Previous studies found that the *ATNRT1.1* regulates abscisic acid (ABA) content, which in turn controls stomatal opening in stemsis and involved in the drought resistance of plants.^39–41^ All apple NRT2 subfamilies promoter have jasmonic acid and abscisic acid motifs.^42^

Co-expression network analyze of B46NRT2.1 was carried out using the TomExpress platforms. Salinity promotes *B46NRT2.1* gene expression in maize B46 lines. If the increased expression of *NRT2.1* in maize was caused by an increase in the activity of another protein under saline conditions, more research is required to confirm this.

## Supplementary Material

Supplemental Material
