# Supplementary material for: Cloning and expression study of a high-affinity nitrate transporter gene from Zea mays L
Source: Plant Signal Behav. 2023 Jan 16;18(1):2163342. doi: 10.1080/15592324.2022.2163342 (PMC9851203; doi:10.1080/15592324.2022.2163342)
Supplement: Supplemental Material [file KPSB_A_2163342_SM8020.docx]

The sequence of gene *B46NRT2.1*

ATGGCGGCCGTCGGCGCTCCGGGCAGCTCTCTGCACGGAGTCACGGGGCGCGAGCCGGCGTTCGCCTTCTCCACGGAGCACGAGGAGGCGGCGAGCAATGGTGGCAAGTTCGACCTGCCGGTGGACTCAGAGCACAAGGCGAAGAGCGTCCGTCTCTTCTCCGTGGCGAACCCACACATGCGCACCTTCCACCTCTCCTGGATCTCCTTCTTCACCTGCTTCGTGTCCACCTTCGCCGCCGCGCCGCTGGTCCCCATCATCCGCGACAACCTCAACCTCACCAAGGCCGACATCGGCAACGCGGGCGTGGCCTCGGTGTCGGGCTCCATCTTCTCCCGCCTCACCATGGGCGCCGTCTGCGACCTGCTGGGCCCGCGCTACGGCTGCGCCTTCCTCATCATGCTGTCCGCGCCCACCGTGTTCTGCATGTCGCTCATCGACGACGCCGCGGGCTACATCACCGTCAGGTTCCTCATCGGCTTCTCCCTCGCCACCTTCGTCTCCTGCCAGTACTGGATGAGCACCATGTTCAGCAGCAAGATCATCGGCACCGTCAACGGGCTCGCCGCCGGATGGGGCACAATGGGAAGGCGGCGCCACGCAGCTCATATGCCGCTCGTCTACGACGTCATCCGCAAGTGCGGCGCCACGCCATTCACGGCCTGGCGCCTCGCCTACTTCGTGCCGGGCCTCATGCACGTCGTCATGGGCGTCCTGGTGCTCACGCTGGGGCAGGACCTCCCCGACGGCAACCTCAGGTCGCTGCAGAAGAAGGGCAACGTCAACAAGGACAGCTTCTCCAAGGTCATGTGGTACGCCGTCATCAACTACCGTACCTGGATCTTTGTCCTCCTCTACGGCTACTGCATGGGCGTCGAGCTCACCACCGACAACGTCATCGCCGAGTACATGTACGACCGCTTCGACCTCGACCTCCGCGTCGCTGGGACCATCGCCGCCTGCTTCGGCATGGCCAACATCGTCGCACGCCCCATGGGCGGCATCATGTCCGACATGGGCGCGCGCTACTGGGGCATGCGCGCTCGCCTCTGGAACATCTGGATCCTCCAGACCGCCGGCGGCGCCTTCTGCCTCTGGCTGGGGCGCGCCAGCACCCTCCCCGTCTCCGTCGTCGCCATGGTGCTCTTCTCCTTCTGCGCGCAGGCGGCATGCGGCGCCATCTTCGGGGTTATCCCCTTTGTCTCCCGCCGCTCCCTCGGCATCATCTCCGGCATGACGGGCGCCGGCGGCAACTTCGGCGCCGGGCTCACGCAGCTGCTCTTCTTTACCTCCTCGACCTACTCCACGGGCAGGGGGCTGGAGTACATGGGCATCATGATCATGGCGTGCACGCTGCCCGTGGTGTTCGTGCACTTCCCTCAGTGGGGGTCCATGTTCTTTCCGCCCAGCGCCACCGCCGACGAGGAGGGC

[Promoter](javascript:;) [Sequence](javascript:;) of *B46NRT2.1*

GAGGGTTTTCTGTACATAGATGATGCTGGCTGGTGCTTGTGCTTGAGGTTCCGGCAATGGAATCGATGGGGGGTACGTATTTATACTTATAGTGAGCTGGGGTGCATGGTTGACATTTTTGGGCATCTCGATATATTATATCAGTTTCTCGATCGAGCGTGGGAGAAGGAGAAGAAAAAGAATTAAAGAAAGATTGAGCTCTGGCGTGTCATGGACATGAAGGGTCGTCGTCAAGCTCAGCTGAGCTTGAGAGCTGAATTAATAAGGCAATTATTGGGTCTGACAGAATCATCGGTGCAGGGTTAATCTTTAGATTGGAGCTAGTAGAACTGGAACAGAGGCTACAAAGAATAGATTAGAGAGAATGCTTGGAGGTTGGCATTTGGTATTAGCCAGCAAAGAAATTAATTAAATGGATTCCTCGGCGCCGCATCACCGGCAGAGGAATTTGACCTTGGGCTTGGCCTCATCCCGTGGGATCAGCCAAGGATCAGAATCTCCTTCCTATCGCAGCTACTAGCTATCCTTGTTGGTGATGCGGCGTCCAGAATCCCGGCAGCAGCAGCCGACTAAACGGCATAGAATAAAAAATGCACATGCCCACCACCAGCCTCCTCCTCCGATCCTCTGCTCTACTGTCACTATTAATCACAAACTTTAGAGACGACTATAATGCCTGCCTCTCTAAATTAGAAGATACAGGACTGAGGAGTCTCTCGAACTTGGCACGCTGTACCACTTGGATCACCGAACGTGAAAAACCATAAAAATGGGTTCTTTAACTTTTCAAAATCGTTCAGATGACATCGCTGCATGCCACCTCGAAGCCTCGCCACACTTGTTGTGTGCCAACTAGTATGTGCAGTCCTCCAAATTTGATATATTGTGTCACTTTGGGTTTTCGAACTTAGTTTGTTGTTTCATTTGGGTCACTAAAATTTAATTAGTGAGCTTCAGCATAGTGCGCTTCAGTATGGTATGGCACGCCACGTCGCATCGATTTGAGTAAA
